# Vaccination opportunity in children up to 6 months old born in 2017 and 2018 in the city of Londrina-PR, Brazil: a population-based survey

**DOI:** 10.1590/S2237-96222024v33e2024432.especial2.en

**Published:** 2025-01-10

**Authors:** Heloísa Dias Brites, Ana Paula França, José Cássio de Moraes, ICV 2020 Group, Adriana Ilha da Silva, Alberto Novaes Ramos, Ana Paula França, Andrea de Nazaré Marvão Oliveira, Antonio Fernando Boing, Carla Magda Allan Santos Domingues, Consuelo Silva de Oliveira, Ethel Leonor Noia Maciel, Ione Aquemi Guibu, Isabelle Ribeiro Barbosa Mirabal, Jaqueline Caracas Barbosa, Jaqueline Costa Lima, José Cássio de Moraes, Karin Regina Luhm, Karlla Antonieta Amorim Caetano, Luisa Helena de Oliveira Lima, Maria Bernadete de Cerqueira Antunes, Maria da Gloria Teixeira, Maria Denise de Castro Teixeira, Maria Fernanda de Sousa Oliveira Borges, Rejane Christine de Sousa Queiroz, Ricardo Queiroz Gurgel, Rita Barradas Barata, Roberta Nogueira Calandrini de Azevedo, Sandra Maria do Valle Leone de Oliveira, Sheila Araújo Teles, Silvana Granado Nogueira da Gama, Sotero Serrate Mengue, Taynãna César Simões, Valdir Nascimento, Wildo Navegantes de Araújo

**Affiliations:** 1 Faculdade de Ciências Médicas da Santa Casa de São Paulo, Acadêmica de Medicina, São Paulo, SP, Brasil; 2 Faculdade de Ciências Médicas da Santa Casa de São Paulo, Departamento de Saúde Coletiva, São Paulo, SP, Brasil; Universidade Federal do Espírito Santo, Vitória, ES, Brazil; Universidade Federal do Ceará, Departamento de Saúde Comunitária, Fortaleza, CE, Brazil; Faculdade Ciências Médicas Santa Casa de São Paulo, São Paulo, SP, Brazil; Secretaria de Estado da Saúde do Amapá, Macapá, AP, Brazil; Universidade Federal de Santa Catarina, SC, Brazil; Organização Pan-Americana da Saúde, Brasília, DF, Brazil; Instituto Evandro Chagas, Belém, PA, Brazil; Faculdade de Ciências Médicas Santa Casa de São Paulo, Departamento de Saúde Coletiva, São Paulo, SP, Brazil; Universidade Federal de Mato Grosso, Cuiabá, MT, Brazil; Universidade Federal do Paraná, Curitiba, PR, Brazil; Universidade Federal de Goiás, Goiânia, GO, Brazil; Universidade Federal do Piauí, Teresina, PI, Brazil; Universidade de Pernambuco, Faculdade de Ciências Médicas, Recife, PE, Brazil; Instituto de Saúde Coletiva, Universidade Federal da Bahia, Salvador, BA, Brazil; Secretaria de Estado da Saúde de Alagoas, Maceió, AL, Brazil; Universidade Federal do Acre, Rio Branco, AC, Brazil; Universidade Federal do Maranhão, Departamento de Saúde Pública, São Luís, MA, Brazil; Universidade Federal de Sergipe, Aracaju, SE, Brazil; Secretaria Municipal de Saúde, Boa Vista, RR, Brazil; Fundação Oswaldo Cruz, Mato Grosso do Sul, Campo Grande, MS, Brazil; Fundação Oswaldo Cruz, Escola Nacional de Saúde Pública Sergio Arouca, Rio de Janeiro, RJ, Brazil; Universidade Federal do Rio Grande do Sul, Porto Alegre, RS, Brazil; Fundação Oswaldo Cruz, Instituto de Pesquisa René Rachou, Belo Horizonte, MG, Brazil; Secretaria de Desenvolvimento Ambiental de Rondônia, Porto Velho, RO, Brazil; Universidade de Brasília, Brasília, DF, Brazil

**Keywords:** Inmunización, Cobertura de Vacunación, Programas de Inmunización, Encuestas Epidemiológicas., Immunization, Vaccination Coverage, Immunization Programs, Health Surveys

## Abstract

**Objective:**

To evaluate opportunity for vaccination in children born alive in Londrina, up to 6 months old and the relationship between socioeconomic stratum and vaccination regularity.

**Method:**

Population survey study based on a retrospective cohort of children born in 2017 and 2018 that identified vaccines not administered in a given session. Vaccination regularity was compared between socioeconomic strata using Pearson’s chi-square test.

**Results:**

Out of 456 vaccination cards, the proportion of vaccination opportunities not recovered for doses to be administered at birth and at two, four and six months was 5.0% (95%CI 3.1;7.5), 4. 5% (95%CI 2.8;6.9), 7.2% (95%CI 5.0;10.2) and 2.1% (95%CI 1.0;4.0), respectively. There was no statistical difference in vaccination regularity between the strata.

**Conclusion:**

Missed opportunities for vaccination were found at all ages. Socioeconomic stratum did not influence vaccination regularity.

## Study contributions

**Main results:**

Excluding doses at birth, more than 80% of children received the recommended doses in the same session for each age. There was no statistical difference between socioeconomic strata in relation to vaccination regularity.

**Implications for services:**

Strategies to address vaccine hesitancy are essential, as well as training health professionals to administer all doses indicated for a given session.

**Perspectives:**

Future studies to investigate the reasons for the need for more than one session to complete the childhood vaccination schedule in Londrina and for missed vaccination opportunities will provide better guidance for public actions.

## RESUMEN

**Objetivo:**

Evaluar la oportunidad de vacunación en niños nacidos vivos en Londrina, hasta los 6 meses de edad y la relación entre el estrato socioeconómico y la regularidad de las vacunaciones.

**Método:**

Estudio de encuesta poblacional basado en una cohorte retrospectiva de personas nacidas en 2017 y 2018 que identificó vacunas no administradas en las sesiones determinadas. La regularidad de la vacunación se comparó entre estratos socioeconómicos mediante la prueba de chi-cuadrado de Pearson.

**Resultados:**

De 456 cartillas de vacunación, la proporción de oportunidades de vacunación no recuperadas para dosis al nacer, a los dos, cuatro y seis meses fue del 5,0% (IC_95_% 3,1;7,5), 4,5% (IC_95_% 2,8;6,9), 7,2% (IC_95_% 5,0;10,2) y 2,1% (IC_95_% 1,0;4,0), respectivamente. No hubo diferencias estadísticas en la regularidad de la vacunación entre los estratos.

**Conclusión:**

Se observaron oportunidades perdidas de vacunación en todas las edades. El estrato socioeconómico no influyó en la regularidad de la vacunación. Palabras clave: Inmunización; Cobertura de Vacunación; Programas de Inmunización; Encuestas Epidemiológicas.

## INTRODUCTION

In Brazil, over the last ten years, there has been a drop in vaccination coverage, especially regarding the childhood schedule.^1^ The inclusion of new vaccines on the routine schedule has made the National Immunization Program (*Programa Nacional de Imunizações* - PNI) more complex in the last two decades, leading to challenges, such as maintaining vaccination coverage,^2^ especially for doses that should be administered in the same session. Sessions are defined for an organized and previously planned period in which immunization services are offered to the population.^3^ During a session, vaccines are administered by health professionals, and sessions can be routine, as part of a regular immunization schedule, or part of vaccination campaigns.^3^ In this context, it is possible to analyze missed vaccination opportunities, which are defined, according to the World Health Organization, as failure to administer partial or total vaccine doses indicated in opportune situations in any contact of the eligible individual with the health system, representing a persistent and significant challenge for global public health.^4^ Opportunities can be considered either to be recovered, i.e. when the vaccines in question are administered later, or unrecovered. Despite the availability of effective and safe vaccines, many people who have contact with health services do not receive recommended immunizations, which contributes to suboptimal vaccination coverage and the reemergence of vaccine-preventable diseases.^1,4,5^ Between 2015 and 2020, Brazil recorded a significant drop in vaccination coverage rates for several diseases.^5^ Poliovirus vaccine, for example, only achieved 75.9% coverage in 2020, while the target set by the PNI is 95%.^5^ This low adherence reflects not only vaccination hesitancy, but also missed opportunities during children’s contact with health services. ^4^

Although these are important obstacles to fulfilling the childhood vaccination schedule, there are still few epidemiological studies that focus on missed vaccination opportunities in recent years in Brazil.^4^ In this context, in Latin America, a systematic review showed missed opportunity rates between 5% and 37%.^6^ In Brazil, a study conducted in the city of Recife showed that almost 40% of vaccination cards were found to have overdue vaccinations as a direct consequence of lost opportunities.^7^ This context points to an urgent need for studies in Brazilian cities to gain a better understanding of missed vaccination opportunities in Brazil. Therefore, the objective of this study is to evaluate vaccination opportunities among children born alive in Londrina, up to 6 months old, and the relationship between socioeconomic stratum and vaccination regularity.

## METHODS

This is a population-based survey based on a retrospective cohort, conducted from October 2021 to January 2022, which verified compliance with the PNI vaccination schedule and factors associated with vaccination regularity. The data were obtained from the National Vaccination Survey (*Inquérito Nacional de Vacinação*).^8^

The study population was made up of children born in 2017 and 2018 living in the city of Londrina, located in the northern region of the state of Paraná, Brazil, where the estimated resident population is 575,377 inhabitants and municipal population density is 306.52 inhab./km^2^. 

The list of newborns in Londrina was georeferenced according to census tract. In the first stage, the census tracts were divided into four strata (A, B, C and D – where A referred to the best socioeconomic conditions and D referred to the poorest), according to socioeconomic data taken from the 2010 Demographic Census. Three indicators were used in order to classify the socioeconomic strata: average income of heads of family, proportion of literate heads of family and proportion of heads of family with monthly income above 20 minimum wages. Thus, using cluster analysis, all census tracts were classified into four strata, according to the combination of these three indicators (Table 1). Once the socioeconomic strata had been established, the number of live births from the 2017 and 2018 cohort potentially living in each census tract was identified, and the tracts were aggregated into clusters containing at least 56 to 65 live births each, in order to obtain a representative sample for each of the strata and facilitate field work. The sample size calculation was made based on estimated coverage of 70%, a 5% estimation error, a 95% confidence level, 80% test power and a design effect of 1.4, due to it being arranged in clusters.

**Table 1:** Characteristics of the socioeconomic strata of the population of the municipality of Londrina-PR, Brazil, for children born in 2017 and 2018 (n = 456)

Strata	Number of census tracts	Average income (BRL)	Literate heads of household (%)	Monthly family income > 20 minimum wages (%)
A	66	5,504.0	99.6	10.8
B	157	2,325.7	98.7	1.3
C	293	1,137.5	94.6	0.1
D	114	657.2	88.5	0.0
Total	630	1,804.1	95.0	1.4

Clusters were formed for each stratum according to the number of live births in each tract. Each socioeconomic stratum was the unit of interest for analysis. However, in order to avoid possible interference of the characteristics of health services in different neighborhoods of the city, the decision was taken to carry out a systematic selection of clusters, so as to have a representative sample of different areas in each of the strata.

In the case of Londrina, in each of the strata four clusters were selected at random and 28 or 29 children living there who were part of the 2017 or 2018 birth cohorts were interviewed. Only one child per household was included.

The full survey questionnaire was composed of nine blocks; however, for this article, we only analyzed variables related to family characteristics (socioeconomic stratum, family consumption level, monthly family income and income transfer program) and variables related to the vaccination process (coincidence of dates of vaccine administration recommended at birth and at 2, 4 and 6 months, and delayed vaccination for any vaccine scheduled up to 6 months old). Family consumption level was defined according to criteria used by the Brazilian Association of Survey Companies (*Associação Brasileira de Empresas de Pesquisa*).^9^

A photographic record of each child’s vaccination card was made in order to check the doses administered; in the case of children who did not have a vaccination card to show to the interviewers, the researchers searched for the corresponding form details on the PNI Information System, and if it was not found, the child in question was considered not to have been vaccinated.

Different vaccines aimed at preventing the same diseases were combined together (for example: diphtheria, tetanus and pertussis [DTP] vaccine; measles, mumps, rubella and varicella [MMRV] vaccine; diphtheria, tetanus, pertussis, *Haemophilus influenzae* b and hepatitis B [DTP-Hib-HepB] vaccine; or DTP-Hib-HepB + inactivated poliovirus vaccine, for the respective diseases), in order to add together the doses administered by both public and private services.^10^

### Study variables 

Coincidence between dose administration dates was defined as being when vaccines scheduled for the same session, according to the age recommended by the PNI schedule, for doses at birth and at 2, 4 and 6 months were administered on the same day. In accordance with the PNI schedule, the following vaccines were considered:

a) At birth: BCG and hepatitis B (HepB);
b) At two months: diphtheria, tetanus, pertussis, *Haemophilus influenzae* B and HepB (DTP-Hib-HepB), inactivated poliovirus 1, 2 and 3 (IPV), rotavirus (RV1) and pneumococcal conjugate (PCV10);
c) At four months: DTP-Hib-HepB, IPV, RV1 and PCV10;
d) At six months: DTP-Hib-HepB and IPV.

When there was no coincidence between vaccine administration dates (missed opportunity), we checked verified whether the doses were administered later (recovered opportunity) or whether they were not administered at all (unrecovered opportunity). In the case of children who did not receive any vaccine, vaccination was considered to be missing.

Considering the sample weights and the study design, the Stata version 17 survey analysis module was used to estimate the proportion of children with doses with coincident dates, those with recovered opportunities and those with one or more doses missing. The precision of these estimates was determined by using 95% confidence intervals (95%CI), while Pearson’s chi-square test with a 5% significance level was used to measure associations with the socioeconomic strata.

Following this, Microsoft Excel was used to analyze unrecovered opportunities and missing vaccinations, while associations were measured by Fisher’s exact test using the OpenEpi application, version 3.01.^11^

The survey was approved by the Research Ethics Committee of the *Instituto de Saúde Coletiva da Universidade Federal da Bahia*, as per Opinion No. 3.366.818, on June 4, 2019, and Certificate of Submission for Ethical Appraisal (*Certificado de Apresentação de Apreciação* Ética - CAAE) No. 4306919.5.0000.5030; and by the Research Ethics Committee of the *Irmandade da Santa Casa de São Paulo*, as per Opinion No. 4.380.019, on November 4, 2020, and CAAE No. 39412020.0.0000.5479.

## RESULTS

Data from 456 children’s vaccination cards were analyzed, comprising 115, 114, 113 and 114 children in socioeconomic strata A, B, C and D, respectively. There was no sample loss. Regarding vaccines scheduled at birth, 6.5% (95%CI 3.5;11.5) of the children received BCG and HepB on the same date, and recovered opportunities reached 78.8% (95%CI 68.6;86.3). In this case, HepB was administered before BCG according to 98.6% (95%CI 96.9;99.6) of the vaccination cards analyzed; vaccination opportunities were not recovered for 5.0% (95%CI 3.1;7.5) of the children, whereby HepB had the lowest coverage and was missing in 86.4% (95%CI 65.0;97.0) of the records. Furthermore, 4.2% (95%CI 2.5;6.4) of children did not receive either vaccine dose at birth.

The proportion of children with coincident doses at birth was higher among those in socioeconomic stratum D (14.1%, 95%CI 6.1;29.2), and those with recovered opportunities, among those in stratum A (85, 0%, 95%CI 68.2;93.6). Stratum B had the highest proportion of missed doses (18.8%, 95%CI 7.0;41.4), however, there was no statistical difference (p=0.180) (Table 2).

**Table 2:** Proportions (%) and 95% confidence intervals (95%CI) of vaccination cards showing recovered opportunities, dates of coincident doses or missing doses of vaccines administered at birth, by socioeconomic strata,in Londrina-PR, Brazil, for children born in 2017 and 2018 (n = 456)

Strata	Doses at birth (BCG-ID and hepatitis B)				p-value
	**Recovered opportunities % (95%CI)**	**Dates of coincident doses % (95%CI)**	**One or more vaccines missing % (95%CI)**	**Total**	
A	84.9 (68.2;93.6)	4.3 (1.0;15.9)	10.8 (4.9;22.2)	100%	
B	79.7 (57.4;91.9)	1.5 (0.4;5.0)	18.8 (7.0;41.4)	100%	
C	80.3 (67.5;88.8)	10.2 (4.7;20.7)	9.6 (4.4;19.6)	100%	
D	69.8 (55.5;81.0)	14.1 (6.1;29.2)	16.1 (4.3;45.0)	100%	
Total	78.8 (68.6;86.3)	6.5 (3.5;11.5)	14.8 (8.2;25.2)	100%	0.180

Regarding doses scheduled to be administered at 2 months, 83.1% (95%CI 67.9;92.0) received all vaccines in the same session, and opportunities were recovered for 11.0% (95%CI 3. 2;31.2) (Table 3). RV1 was the vaccine with the highest proportion of recovered opportunities (48.5%, 95%CI 30.8;66.46). Furthermore, opportunities were not recovered for 4.5% (95%CI 2.8;6.9) of the children, who received one to three doses. Among them, 70% (95%CI 45.7;88.1) received three doses; 25% (95%CI 8.6;49.1) only received two doses; and 5% (95%CI 0.1;24.9) only received one vaccine. RV1 had the lowest coverage among children with an incomplete vaccination schedule, being missing in 60% (95%CI 36.0;80.8) of these cases; 3.7% (95%CI 2.1;5.9) of the children did not receive any vaccine.

**Table 3:** Proportions (%) and 95% confidence intervals (95%CI) of vaccination cards showing recovered opportunities, dates of coincident doses or missing doses of vaccines administered at 2 months, by socioeconomic strata, in Londrina- Paraná, Brazil, for children born in 2017 and 2018 (n = 456)

Strata	Doses at two months (DTP-Hib-HepB, IPV, rotavirus and PVC10)				p-value
	**Recovered opportunities % (95%CI)**	**Dates of coincident doses % (95%CI)**	**One or more vaccines missing % (95%CI)**	**Total**	
A	7.6 (4.4;12.9)	81.7 (72.1;88.6)	10.7 (4.6;22.7)	100%	
B	18.4 (3.9;55.3)	78.9 (45.6;94.4)	2.6 (0.80;8.4)	100%	
C	4.8 (1.9;11.5)	85.5 (77.0;91.1)	9.7 (5.0;18.2)	100%	
D	4.2 (1.4;11.8)	91.0 (79.3;96.4)	4.8 (1.8;12.2)	100%	
Total	11.0 (3.2;31.2)	83.1 (67.9;92.0)	5.9 (3.2;10.7)	100%	0.083

Still with regard to doses administered at 2 months, the proportion of children with coincident doses was higher among those in socioeconomic stratum D (91.0%, 95%CI79.3;96.4), and those with recovered opportunities, among those in stratum B (18.4%, 95%CI 3.9;55.3). Stratum A had the highest proportion of missing vaccines (10.7%, 95%CI 4.6;22.7). There was no statistical difference (p=0.083) (Table 3).

Regarding the doses scheduled to be administered at 4 months, 82.0% (95%CI 72.3;88.9) received all vaccines in the same session and 4.6% (95%CI 2.4;8.7) had opportunities recovered (Table 4). RV1 had the most recovered opportunities, and was administered late among 48.6% (95%CI 31.92;65.6). Furthermore, opportunities were not recovered for 7.2% (95%CI 5.0;10.2) of the children, who received between one and three doses. Among these children, 68.7% (95%CI 50.0;83.9) received three doses; 22.0% (95%CI 9.3;40.0) only received two doses; and 9.3% (95%CI 2.0;25.0) only received one dose. RV1 had the lowest coverage among children with an incomplete vaccination schedule, being missing on 72.0% (95%CI 53.2;86.2) of the vaccination cards; 4.2% (95%CI 2.5;6.4) of the children did not receive any vaccine.

**Table 4:** Proportions (%) and 95% confidence intervals (95%CI) of vaccination cards showing recovered opportunities, dates of coincident doses or missing doses of vaccines administered at 4 months, by socioeconomic strata, in Londrina-PR, Brazil, for children born in 2017 and 2018 (n = 456)

Strata	Doses at four months (DTP-Hib-HepB, IPV, rotavirus and PCV10)				
	**Recovered opportunities** **% (95%CI)**	**Dates of coincident doses % (95%CI)**	**One or more vaccines missing** **% (95%CI)**	**Total**	**p-value**
A	9.9 (4.2;21.7)	79.0 (66.7;87.6)	11.0 (4.9;23.0)	100%	
B	1.0 (0.3;3.5)	83.0 (60.2;94.0)	16.0 (5.3;39.3)	100%	
C	8.9 (4.0;18.5)	77.6 (66.9;85.6)	13.5 (6.0;27.6)	100%	
D	3.0 (1.1;7.7)	90,2 (80.0;95.5)	6,8 (3.0;14.8)	100%	
Total	4.6 (2.4;8.7)	82.0 (72.3;88.9)	13.3 (7.1;23.8)	100%	0.178

The proportion of children with coincident doses at 4 months was higher among those in socioeconomic stratum D (90.2%, 95%CI 80.0;95.5), and those with recovered opportunities, among those in stratum A (9.9%, 95%CI 4.2;21.7). Stratum B had the highest proportion of missing vaccines (16.0%, 95%CI 5.3;39.3), however, there was no statistical difference (p=0.178) (Table 4).

Finally, regarding vaccines scheduled at 6 months old, 83.0% (95%CI 73.6;89.6) received all doses on the same date, and opportunities were recovered for 4.8% (95%CI 2.0;10.7) of them (Table 5). In these cases, DTP-Hib-HepB was administered before IPV in 50.0% (95%CI 28.2;72.0) of cases; opportunities were not recovered for 2.1% (95%CI 1.0;4.0) of the children, whereby IPV had the lowest coverage, being missing for 66.7% (95%CI 30.0;92.5) of these children. Moreover, 7.4% (95%CI 5.2;10.2) of the children did not receive either dose.

**Table 5:** Proportions (%) and 95% confidence intervals (95%CI) of vaccination cards showing recovered opportunities, dates of coincident doses or missing doses of vaccines administered at 6 months, by socioeconomic strata, in Londrina-PR, Brazil, for children born in 2017 and 2018 (n = 456)

Strata	Doses at six months (DTP-Hib-HepB and IPV)				**p-value**
	**Recovered opportunities % (95%CI)**	**Dates of coincident doses % (95%CI)**	**One or more vaccines missing** **% (95%CI)**	**Total**	
A	4.3 (1.9;9.5)	82.3 (72.0;89.4)	13.3 (6.5;25.4)	100%	
B	3.4 (0.5;19.8)	81.1 (58.6;92.9)	15.5 (5.0;39.2)	100%	
C	8.1 (3.3;18.9)	80.9 (73.8;86.4)	10.9 (5.0;22.4)	100%	
D	2.0 (0.6;6.8)	93.3 (82.8;97.6)	4.7 (1.3;15.8)	100%	
Total	4.8 (2.0;10.7)	83.0 (73.6;89.6)	12.2 (6.2;22.6)	100%	0.499

Still regarding doses to be administered at 6 months, socioeconomic stratum D showed greater coincidence between the vaccine administration dates (93.3%, 95%CI 82.8;97.6), stratum C had more recovered opportunities (8.1%, 95%CI 3.3;18.9), while stratum B had the highest proportion of missing vaccines (15.5%, 95%CI 5.0;39.2). There was no statistical significance (p=0.499) (Table 5).

## DISCUSSION

Regarding missed opportunities for vaccination that were not recovered, the proportions varied according to the age group studied. The highest values were found for vaccines administered at four months and at birth, while recovered opportunities occurred for doses administered at birth and at two months. There was no statistical difference in the relationship between socioeconomic strata and vaccination regularity.

In the case of vaccines administered at birth, BCG had a higher proportion of recovered vaccination opportunities. Late BCG vaccination demonstrates that it is not being administered in maternity wards, as recommended by the Ministry of Health. As administering this vaccine involves a specific and complex technique,^3^ lack of technical training is one of the main causes for it not being administered to newborns, as pointed out by a study conducted in the city of Porto Alegre.^12^ Notwithstanding, HepB vaccine showed the highest rate of unrecovered opportunities, which was also found in an observational study involving children under 1 year old in São Paulo.^13^ Failure to apply HepB in the first 12 hours of life is extremely serious, as this window of time is crucial for preventing vertical transmission of the virus from mother to baby, if the mother has HepB. When the vaccine is administered within this critical period, it acts as an immediate prophylaxis measure, preventing infection before the virus has the opportunity to establish itself in the child’s body.^3^

Regarding vaccines administered at two and four months, RV1 had the highest proportion of unrecovered opportunities. A retrospective study conducted in Peru using a national database also highlights this result and warns of its growing trend in South America.^14^ RV1 is of great importance for public health, drastically reducing the rates of hospitalizations, complications and deaths from the disease.^15^ The first dose of RV1 should be given up to three and a half months of age, and the second dose at seven and a half months. This rigid limit on the age at which the vaccine can be administered may explain the considerable number of missed vaccination opportunities that were not recovered.^16,17^ A study conducted in the United States also indicates parental concern about the overall safety of the vaccine and shortcomings in vaccination services as the main causes that contribute to the drop in RV1 coverage, in addition to showing that the majority of unvaccinated children had at least one missed vaccination opportunity, indicating that there would have been a 10% increase in full RV1 vaccination if all these opportunities had not been missed.^18,19^

Regarding vaccines administered at six months, the third doses of DTP-Hib-HepB and IPV did not show such considerable losses when compared to the previous doses. IPV had the highest proportion of recovered and unrecovered opportunities, differently to what was found in a study conducted in the city of São Luís.^20^ On the other hand, lack of DTP-Hib-HepB and IPV vaccination is consistent with a global trend of falling coverage of both vaccines, and is similar to the findings of articles that analyzed vaccination rates at national and regional levels in recent years.^5,21^

Recovered vaccination opportunities, despite enabling children to receive the vaccines they missed, indicate that they did not receive the vaccines at the right time, requiring more visits to health services in order to complete the vaccination schedule. As recovered opportunities enable detection of irregularities in the administration of a given vaccine, identifying them is extremely important for preventing the emergence of shortcomings in child immunization. In our study, with the exception of doses administered at birth, at all the other ages there was a vaccine with the greatest recovered opportunity as well as a vaccine with the greatest missed opportunity. Furthermore, recovered opportunities do not necessarily imply valid vaccine doses, as each vaccine has a minimum period in which it must be administered in order to achieve a better immune response.^3^

The reported causes of missed vaccination opportunities are currently varied and common across several published articles. In studies carried out in Cape Town (South Africa), Nigeria, Malawi and Chad, using questionnaires for parents and checking vaccination cards when leaving health services, the number of missed vaccination opportunities is much higher than those found in Londrina, reaching rates of almost 40%.^22-24^ Taking children aged 0 to 23 months, they reinforce the large proportion of incomplete vaccinations among children under 1 year old, indicating the main causes to be lack of checking vaccination cards, lack of health worker knowledge and training on the subject, parents’ lack of knowledge about the importance of vaccines and missed opportunities for vaccination, and problems with vaccination campaigns and the distribution and supply of vaccines. These causes coincide with those highlighted by the study conducted in Peru,^14^ which further adds as causes fear of adverse effects and refusal to be vaccinated with a large number of vaccines simultaneously. The latter may even be associated with recovered opportunities. According to a study conducted in Argentina,^25^ making the most of all opportunities for simultaneous administration of all age-eligible doses of vaccines, during the same vaccination visit, is a crucial strategy for achieving vaccination coverage targets. Therefore, it is essential to train health workers both to administer vaccines and to check children’s vaccination cards in any contact they have with health services, so as to address missing vaccines. 

The literature shows that socioeconomic class, parent and guardian literacy level and cultural factors directly influence adherence to the childhood vaccination schedule.^4,10,25,26^ Socioeconomic stratification had the advantage of enabling adequate representation of the different census tracts in Londrina, offering a clear view of unequal vaccination.

Socioeconomic stratum D – that is, children living in the most disadvantaged census tracts – had the highest proportions of coincident dates among doses received at their respective ages when compared to other strata, while strata A and C had the lowest levels of missed vaccination. However, there was no statistical difference between the strata. 

In surveys in Brazilian state capitals, children from higher socioeconomic strata had the lowest full vaccination rate and lower coverage for several individual vaccines,^26^ demonstrating a contemporary trend of falling coverage that is related to the wealthiest strata. This suggests that public service immunization programs, which are the most used by the most disadvantaged population, are fulfilling PNI recommendations, and that vaccination losses are probably not related to the structure of these vaccination services.

Over the last 40 years, the decrease in the occurrence of vaccine-preventable diseases linked to growing waves of false information about the adverse effects of vaccines may have led to an attitude of disinterest and hesitation among the most privileged segments of Londrina’s population, as already reported in high-income countries and in Brazil.^27-29^ This scenario leads to the childhood vaccination schedule not being adhered to by high socioeconomic strata, causing vaccination loss rates. As most of these children tend to use private vaccination services, it is important to understand the dynamics adopted by health professionals, in order to minimize the hesitancy of parents and guardians who prevent children from receiving all the necessary vaccines in a given session. As such, it is essential that public authorities identify the main barriers that make it difficult for the higher strata population to regularize their children’s vaccination schedule, and to target educational campaigns that demystify the fears of this segment of the population. Furthermore, there is a need to involve the private health system in addressing missed vaccination opportunities.

The study encompasses a significant distribution of children in the urban area of Londrina, forming a representative sample of that area, in addition to their being no sample losses. Despite this, as a limitation, it did not cover the rural area, which has a population of 13,181 children.^30^ Furthermore, reading vaccination cards may also lead to errors, given the lack of standardization of notes and illegible dates, making data transcription difficult. 

The study reveals vaccination losses for vaccines that should be administered in the same session. Therefore, educational campaigns focused on misinformation and fear of adverse effects, training of vaccination session professionals and better integration between public and private health systems are recommended, in order to guarantee continuous monitoring of vaccination status. Future studies should explore the causes of vaccine hesitancy and strategies to reduce these missed opportunities, with the aim of achieving greater adherence to the vaccination schedule.
