# Supplementary material for: Vaccination opportunity in children up to 6 months old born in 2017 and 2018 in the city of Londrina-PR, Brazil: a population-based survey
Source: Epidemiol Serv Saude. 2025 Jan 10;33(spe2):e2024432. doi: 10.1590/S2237-96222024v33e2024432.especial2.en (PMC11734602; doi:10.1590/S2237-96222024v33e2024432.especial2.en)
Supplement: Translation

# Translation: Oportunidade vacinal em crianças de até 6 meses, nascidas em 2017 e 2018 no município de Londrina: inquérito de base populacional

**DOI:** 10.1590/S2237-96222024v33e2024432.especial2.pt

**Authors:** Heloísa Dias Brites, Ana Paula França, José Cássio de Moraes, Grupo ICV 2020, Adriana Ilha da Silva, Alberto Novaes Ramos, Ana Paula França, Andrea de Nazaré Marvão Oliveira, Antonio Fernando Boing, Carla Magda Allan Santos Domingues, Consuelo Silva de Oliveira, Ethel Leonor Noia Maciel, Ione Aquemi Guibu, Isabelle Ribeiro Barbosa Mirabal, Jaqueline Caracas Barbosa, Jaqueline Costa Lima, José Cássio de Moraes, Karin Regina Luhm, Karlla Antonieta Amorim Caetano, Luisa Helena de Oliveira Lima, Maria Bernadete de Cerqueira Antunes, Maria da Gloria Teixeira, Maria Denise de Castro Teixeira, Maria Fernanda de Sousa Oliveira Borges, Rejane Christine de Sousa Queiroz, Ricardo Queiroz Gurgel, Rita Barradas Barata, Roberta Nogueira Calandrini de Azevedo, Sandra Maria do Valle Leone de Oliveira, Sheila Araújo Teles, Silvana Granado Nogueira da Gama, Sotero Serrate Mengue, Taynãna César Simões, Valdir Nascimento, Wildo Navegantes de Araújo

**Affiliations:** 1 Faculdade de Ciências Médicas da Santa Casa de São Paulo, Acadêmica de Medicina, São Paulo, SP, Brasil; 2 Faculdade de Ciências Médicas da Santa Casa de São Paulo, Departamento de Saúde Coletiva, São Paulo, SP, Brasil; Universidade Federal do Espírito Santo, Vitória, ES, Brasil; Universidade Federal do Ceará, Departamento de Saúde Comunitária, Fortaleza, CE, Brasil; Faculdade Ciências Médicas Santa Casa de São Paulo, São Paulo, SP, Brasil; Secretaria de Estado da Saúde do Amapá, Macapá, AP, Brasil; Universidade Federal de Santa Catarina, SC, Brasil; Organização Pan-Americana da Saúde, Brasília, DF, Brasil; Instituto Evandro Chagas, Belém, PA, Brasil; Faculdade de Ciências Médicas Santa Casa de São Paulo, Departamento de Saúde Coletiva, São Paulo, SP, Brasil; Universidade Federal de Mato Grosso, Cuiabá, MT, Brasil; Universidade Federal do Paraná, Curitiba, PR, Brasil; Universidade Federal de Goiás, Goiânia, GO, Brasil; Universidade Federal do Piauí, Teresina, PI, Brasil; Universidade de Pernambuco, Faculdade de Ciências Médicas, Recife, PE, Brasil; Instituto de Saúde Coletiva, Universidade Federal da Bahia, Salvador, BA, Brasil; Secretaria de Estado da Saúde de Alagoas, Maceió, AL, Brasil; Universidade Federal do Acre, Rio Branco, AC, Brasil; Universidade Federal do Maranhão, Departamento de Saúde Pública, São Luís, MA, Brasil; Universidade Federal de Sergipe, Aracaju, SE, Brasil; Secretaria Municipal de Saúde, Boa Vista, RR, Brasil; Fundação Oswaldo Cruz, Mato Grosso do Sul, Campo Grande, MS, Brasil; Fundação Oswaldo Cruz, Escola Nacional de Saúde Pública Sergio Arouca, Rio de Janeiro, RJ, Brasil; Universidade Federal do Rio Grande do Sul, Porto Alegre, RS, Brasil; Fundação Oswaldo Cruz, Instituto de Pesquisa René Rachou, Belo Horizonte, MG, Brasil; Secretaria de Desenvolvimento Ambiental de Rondônia, Porto Velho, RO, Brasil; Universidade de Brasília, Brasília, DF, Brasil

**Keywords:** Imunização, Cobertura Vacinal, Programas de Imunização, Inquéritos Epidemiológicos

## Abstract

**Objetivo:**

Avaliar a oportunidade vacinal em crianças nascidas vivas em Londrina, com até 6 meses de idade, e a relação do estrato socioeconômico com a regularidade das vacinas.

**Método:**

Estudo de inquérito populacional baseado em uma coorte retrospectiva de nascidos em 2017 e 2018, que identificou vacinas não administradas nas sessões determinadas. A regularidade vacinal foi comparada entre os estratos socioeconômicos pelo teste qui-quadrado de Pearson.

**Resultados:**

Em 456 carteiras de vacinação, a proporção de oportunidades de vacinação não recuperadas para as doses ao nascer, aos 2, 4 e 6 meses foi de 5,0% (IC_95_% 3,1;7,5), 4,5% (IC_95_% 2,8;6,9), 7,2% (IC_95_% 5,0;10,2) e 2,1% (IC_95_% 1,0;4,0), respectivamente. Não houve diferença estatística entre os estratos na regularidade vacinal.

**Conclusão:**

Foram observadas oportunidades perdidas de vacinação em todas as idades. O estrato socioeconômico não influenciou a regularidade vacinal.

## Contribuições do estudo

**Principais resultados:**

Excluindo-se as doses ao nascer, mais de 80% das crianças receberam as doses previstas na mesma sessão para cada idade. Não houve diferença estatística entre os estratos socioeconômicos na relação com a regularidade vacinal.

**Implicações para os serviços:**

Estratégias de combate à hesitação vacinal são essenciais, bem como a capacitação dos profissionais de saúde para aplicação de todas as doses indicadas para determinada sessão.

**Perspectivas:**

Estudos futuros que investiguem os motivos da necessidade de mais de uma sessão para completar o esquema vacinal infantil em Londrina e das oportunidades perdidas de vacinação trarão melhor direcionamento para as ações públicas.

## INTRODUÇÃO

No Brasil, nos últimos dez anos, observou-se uma queda na cobertura vacinal, principalmente no calendário infantil.^1^ A inclusão de novas vacinas no calendário de rotina tornou o Programa Nacional de Imunizações (PNI) mais complexo nas últimas duas décadas, gerando desafios, como manter altas coberturas vacinais,^2^ principalmente para doses que devem ser aplicadas na mesma sessão. Esta é definida por um período organizado e previamente planejado em que os serviços de imunização são oferecidos à população.^3^ Durante uma sessão, as vacinas são administradas por profissionais de saúde, e ela pode ser de rotina, como parte de um calendário regular de imunizações, ou parte de campanhas de vacinação.^3^ Nesse contexto, é possível analisar as oportunidades perdidas de vacinação, as quais são definidas, segundo a Organização Mundial da Saúde, como a não administração parcial ou total de doses vacinais indicadas em situações oportunas em qualquer contato do indivíduo elegível com o sistema de saúde, representando um desafio persistente e significativo para a saúde pública global,^4^ podendo a oportunidade ser considerada recuperada, quando tais vacinas são aplicadas posteriormente, ou não recuperada. Apesar da disponibilidade de vacinas eficazes e seguras, muitas pessoas que têm contato com os serviços de saúde não recebem as imunizações recomendadas, o que contribui para coberturas vacinais abaixo do ideal e para a reemergência de doenças imunopreveníveis.^1,4,5^ Entre 2015 e 2020, o Brasil registrou uma queda significativa nas taxas de cobertura vacinal para diversas doenças.^5^ A vacina contra a poliomielite, por exemplo, alcançou apenas 75,9% de cobertura em 2020, sendo a meta estabelecida pelo PNI de 95%.^5^ Essa baixa adesão reflete não apenas a hesitação vacinal, mas também as oportunidades perdidas durante o contato das crianças com os serviços de saúde. ^4^

Apesar de elas serem obstáculos importantes para o cumprimento do calendário vacinal infantil, ainda há poucos estudos epidemiológicos que se debruçam sobre as oportunidades perdidas de vacinação nos últimos anos no país.^4^ Nesse contexto, na América Latina, uma revisão sistemática apontou taxas de perdas entre 5% e 37%.^6^ No país, um estudo realizado em Recife mostrou que quase 40% das carteiras de vacinação apresentavam atraso vacinal como consequência direta dessas oportunidades perdidas.^7^ Esse contexto aponta para uma necessidade urgente de estudos nas cidades brasileiras para melhor compreensão das perdas vacinais no país. Por isso, este estudo se dedica a avaliar a oportunidade vacinal em crianças nascidas vivas em Londrina, com até 6 meses de idade, e a relação do estrato socioeconômico com a regularidade das vacinas.

## MÉTODOS

Trata-se de inquérito de base populacional baseado em coorte retrospectiva, realizada de outubro de 2021 a janeiro de 2022, que verificou o cumprimento do calendário vacinal do PNI e os fatores associados à regularidade de vacinação. Os dados foram obtidos do Inquérito Nacional de Vacinação.^8^

A população do estudo foi composta por crianças nascidas em 2017 e 2018 residentes do município de Londrina, localizado na região norte do Paraná, onde a população residente estimada é de 575.377 habitantes e a densidade demográfica municipal é de 306,52 hab./km^2^. 

A lista dos recém-nascidos de Londrina foi georreferenciada por setor censitário. Na primeira etapa, os setores foram divididos em quatro estratos (A, B, C e D – sendo A o de melhor e D o de pior condição socioeconômica), segundo dados socioeconômicos do Censo Demográfico de 2010. Para a construção dos estratos socioeconômicos, foram utilizados três indicadores: renda média dos responsáveis pelas famílias, proporção de responsáveis alfabetizados e proporção dos responsáveis com renda mensal superior a 20 salários mínimos. Assim, utilizando-se uma análise de *cluster*, todos os setores censitários foram classificados em quatro estratos, de acordo com a combinação desses três indicadores (Tabela 1). Estabelecidos os estratos socioeconômicos, foi identificado o número de nascidos vivos da coorte de 2017 e 2018 potencialmente residentes em cada setor censitário, e estes setores foram agregados em conglomerados contendo pelo menos 56 a 65 nascidos vivos cada um, de modo a se obter uma amostra representativa em cada um dos estratos e facilitar o trabalho de campo. O cálculo do tamanho da amostra foi feito a partir de uma cobertura estimada de 70%, com erro de estimativa de 5%, nível de confiança de 95%, poder do teste de 80% e efeito do desenho de 1,4, por conta do arranjo em conglomerados.

**Tabela 1:** Características dos estratos socioeconômicos da população do município de Londrina, para nascidos entre 2017 e 2018 (n = 456)

Estratos	Número de setores	Renda média (R$)	Responsáveis alfabetizados (%)	Renda familiar mensal > 20 salários mínimos (%)
A	66	5.504,0	99,6	10,8
B	157	2.325,7	98,7	1,3
C	293	1.137,5	94,6	0,1
D	114	657,2	88,5	0,0
Total	630	1.804,1	95,0	1,4

Em cada um dos estratos se formaram conglomerados de acordo com o número de nascidos vivos de cada setor. A unidade de interesse para a análise foi o estrato socioeconômico, mas, para evitar a possível interferência das características dos serviços de saúde em diferentes bairros da cidade, optou-se por realizar o sorteio sistemático de conglomerados, de modo a termos uma amostra representativa das diferentes áreas em cada um dos estratos.

No caso de Londrina, em cada um dos estratos foram sorteados quatro conglomerados e entrevistadas 28 a 29 crianças neles residentes e que pertencessem às coortes de nascimento de 2017 ou 2018. Em cada domicílio, apenas uma criança foi incluída.

O questionário completo da pesquisa foi composto por nove blocos; porém, para este artigo, foram analisadas variáveis relacionadas às características da família (estrato socioeconômico, nível de consumo familiar, renda mensal familiar e programa de transferência de renda) e do processo de vacinação (coincidência de datas das vacinas recomendadas ao nascer, aos 2, 4 e 6 meses, e atraso vacinal em qualquer vacina prevista até os 6 meses). O nível de consumo das famílias foi definido segundo critério da Associação Brasileira de Empresas de Pesquisa.^9^

Para verificação das doses aplicadas, foi obtido o registro fotográfico da caderneta de vacinação; para a criança que não tinha caderneta disponível, buscou-se a ficha espelho no Sistema de Informações do PNI, e, quando esta não foi encontrada, a criança foi considerada não vacinada.

Foi realizada a junção de diferentes vacinas voltadas para a prevenção das mesmas doenças (por exemplo, vacina tríplice bacteriana [difteria, tétano e pertússis, DTP], tetravalente, pentavalente ou hexavalente para as respectivas doenças), a fim de serem somadas as doses aplicadas pelo setor público e por serviços privados.^10^

### Variáveis de estudo

A coincidência entre as datas de aplicação das doses foi definida quando vacinas previstas para uma mesma sessão, de acordo com a idade recomendada no calendário do PNI, para as doses ao nascer, 2, 4 e 6 meses foram aplicadas no mesmo dia. Foram consideradas, segundo o PNI, as seguintes vacinas:

a) Ao nascer: BCG e hepatite B (HepB);
b) Dois meses: difteria, tétano, pertússis, HepB e *Haemophilus influenzae* B (penta), poliomielite 1, 2 e 3 inativada (VIP), rotavírus humano (VRH) e pneumocócica 10-valente (Pneumo-10);
c) Quatro meses: penta, VIP, VRH e pneumo 10;
d) Seis meses: penta e VIP.

Quando não houve coincidência entre as datas de aplicação das vacinas (oportunidade perdida), verificou-se se as doses foram aplicadas posteriormente (oportunidade recuperada) ou se não foram aplicadas (oportunidade não recuperada). Crianças que não receberam nenhuma vacina foram consideradas como vacinação ausente.

Considerando-se os pesos amostrais e o delineamento do estudo, foi utilizado o *software Stata* versão 17, no módulo *survey analysis*, para estimar a proporção de crianças com doses com datas coincidentes, com oportunidades recuperadas e a ausência de uma ou mais doses. A precisão dessas estimativas foi feita pelos intervalos de confiança de 95% (IC_95_%) e, para medir associações com o estrato socioeconômico, o teste qui-quadrado de Pearson, adotando-se nível de significância de 5%.

Posteriormente, para analisar as oportunidades não recuperadas e a vacinação ausente, os dados foram analisados no Microsoft Excel, e as associações medidas pelo teste exato de Fisher, no aplicativo OpenEpi, versão 3.01.^11^

O estudo foi aprovado pelos Comitês de Ética em Pesquisa com Seres Humanos do Instituto de Saúde Coletiva da Universidade Federal da Bahia, sob parecer n^o^ 3.366.818, em 4 de junho de 2019, com Certificado de Apresentação de Apreciação Ética (CAAE) 4306919.5.0000.5030; e da Irmandade da Santa Casa de São Paulo, sob parecer n^o^ 4.380.019, em 4 de novembro de 2020, com CAAE 39412020.0.0000.5479.

## RESULTADOS

Os dados das cadernetas de 456 crianças foram analisados e, nos estratos socioeconômicos A, B, C e D, havia, respectivamente, 115, 114, 113 e 114 crianças, e não se verificou perda amostral. Com relação às vacinas previstas ao nascer, 6,5% (IC_95_% 3,5;11,5) receberam BCG e HepB na mesma data, e houve 78,8% (IC_95_% 68,6;86,3) de oportunidades recuperadas. Neste caso, a HepB foi aplicada antes da vacina BCG em 98,6% (IC_95_% 96,9;99,6) das carteiras analisadas; 5,0% (IC_95_% 3,1;7,5) das crianças tiveram oportunidades não recuperadas, sendo a HepB a vacina de menor cobertura, ausente em 86,4% (IC_95_% 65,0;97,0). E 4,2% (IC_95_% 2,5;6,4) das crianças não receberam nenhuma das duas doses.

A proporção de crianças com doses coincidentes ao nascer foi maior entre as do estrato socioeconômico D (14,1%, IC_95_% 6,1;29,2), e as de oportunidades recuperadas, entre as do estrato A (85,0%, IC_95_% 68,2;93,6). O estrato B apresentou a maior proporção de doses perdidas (18,8%, IC_95_% 7,0;41,4), porém, não houve diferença estatística (p=0,180) (Tabela 2).

**Tabela 2:** Tabela 2 – Proporções (%) e intervalos de confiança de 95% (IC _95_) das carteiras de vacinação com oportunidades recuperadas, datas das doses coincidentes ou doses ausentes referentes às vacinas aplicadas ao nascer, por estrato socioeconômico, em Londrina, nascidos em 2017 e 2018 (n = 456)

Estrato	Doses ao nascer (BCGID e hepatite B)				p-valor
	**Oportunidades** **recuperadas** **% (IC**_95_ **%** **)**	**Datas das** **doses coincidentes** **% (IC**_95_ **%** **)**	**Ausência** **de uma ou mais vacinas** **% (IC**_95_ **%** **)**	**Total**	
A	84,9 (68,2;93,6)	4,3 (1,0;15,9)	10,8 (4,9;22,2)	100%	
B	79,7 (57,4;91,9)	1,5 (0,4;5,0)	18,8 (7,0;41,4)	100%	
C	80,3 (67,5;88,8)	10,2 (4,7;20,7)	9,6 (4,4;19,6)	100%	
D	69,8 (55,5;81,0)	14,1 (6,1;29,2)	16,1 (4,3;45,0)	100%	
Total	78,8 (68,6;86,3)	6,5 (3,5;11,5)	14,8 (8,2;25,2)	100%	0,180

Por seu turno, em relação às doses previstas para serem aplicadas aos 2 meses, 83,1% (IC_95_% 67,9;92.0) receberam todas as vacinas na mesma sessão, e 11,0% (IC_95_% 3,2;31,2) tiveram oportunidades recuperadas (Tabela 3). A VRH foi a vacina com maior proporção de oportunidade recuperada (48,5%, com IC_95_% 30,8;66,46). Ainda, 4,5% (IC_95_% 2,8;6,9) das crianças tiveram oportunidades não recuperadas, recebendo de uma a três doses. Dessas, 70% (IC_95_% 45,7;88,1) receberam três doses; 25% (IC_95_% 8,6;49,1) receberam somente duas doses; e 5% (IC_95_% 0,1;24,9), uma só vacina. A vacina VRH foi a de menor cobertura entre as crianças com esquema incompleto de vacinação, ausente em 60% (IC_95_% 36,0;80,8) desses casos; 3,7% (IC_95_% 2,1;5,9) das crianças não receberam nenhuma vacina.

**Tabela 3:** Proporções (%) e intervalos de confiança de 95% (IC_95_) das carteiras de vacinação com oportunidades recuperadas, datas das doses coincidentes ou doses ausentes referentes às vacinas aplicadas aos 2 meses, por estrato socioeconômico, em Londrina, de nascidos em 2017 e 2018 (n = 456)

Estrato	Doses aos dois meses (pentavalente, VIP, rotavírus e pneumocócica)				**p-valor**
	**Oportunidades recuperadas** **% (IC**_95_ **%** **)**	**Datas das** **doses coincidentes** **% (IC**_95_ **%** **)**	**Ausência** **de uma ou mais vacinas** **% (IC**_95_ **%** **)**	**Total**	
A	7,6 (4,4;12,9)	81,7 (72,1;88,6)	10,7 (4,6;22,7)	100%	
B	18,4 (3,9;55,3)	78,9 (45,6;94,4)	2,6 (0,80;8,4)	100%	
C	4,8 (1,9;11,5)	85,5 (77,0;91,1)	9,7 (5,0;18,2)	100%	
D	4,2 (1,4;11,8)	91,0 (79,3;96,4)	4,8 (1,8;12.2)	100%	
Total	11,0 (3,2;31,2)	83,1 (67,9;92,0)	5,9 (3,2;10,7)	100%	0,083

Para as doses aplicadas aos 2 meses, a proporção de crianças com doses coincidentes foi maior entre as do estrato socioeconômico D (91,0%, IC_95_%79,3;96.4), e as de oportunidades recuperadas, entre as do estrato B (18,4%, IC_95_% 3,9;55,3). O estrato A apresentou a maior proporção de ausência de vacinas (10,7%, IC_95_% 4,6;22,7). Não houve diferença estatística (p=0,083). (Tabela 3)

Em relação às doses previstas para serem aplicadas aos 4 meses, 82,0% (IC_95_% 72,3;88,9) receberam todas as vacinas na mesma sessão e 4,6% (IC_95_% 2,4;8,7) tiveram oportunidades recuperadas (Tabela 4). A VRH foi a vacina de maior oportunidade recuperada, atrasada em 48,6% (IC_95_% 31,92;65,6). Ainda, 7,2% (IC_95_% 5,0;10,2) das crianças tiveram oportunidades não recuperadas, e receberam entre uma e três doses. Dessas, 68,7% (IC_95_% 50,0;83,9) receberam três doses; 22,0% (IC_95_% 9,3;40,0) receberam somente duas; e 9,3% (IC_95_% 2,0;25,0), uma só vacina. A vacina VRH foi a de menor cobertura entre as crianças com esquema incompleto de vacinação, ausente em 72,0% (IC_95_% 53,2;86,2) das carteiras; 4,2% (IC_95_% 2,5;6,4) das crianças não receberam nenhuma vacina.

**Tabela 4:** Proporções (%) e intervalos de confiança de 95% (IC_95_) das carteiras de vacinação com oportunidades recuperadas, datas das doses coincidentes ou doses ausentes referentes às vacinas aplicadas aos 4 meses, por estrato socioeconômico, em Londrina, de nascidos em 2017 e 2018 (n = 456)

Estrato	Doses aos quatro meses (penta, VIP, rotavírus e pneumocócica)				**p-valor**
	**Oportunidades recuperadas** **% (IC**_95_ **%** **)**	**Datas das** **doses coincidentes** **% (IC**_95_ **%** **)**	**Ausência** **de uma ou mais vacinas** **%** **(IC**_95_ **%** **)**	**Total**	
A	9,9 (4,2;21,7)	79,0 (66,7;87,6)	11,0 (4,9;23,0)	100%	
B	1,0 (0,3;3,5)	83,0 (60,2;94,0)	16,0 (5,3;39,3)	100%	
C	8,9 (4,0;18,5)	77,6 (66,9;85,6)	13,5 (6,0;27,6)	100%	
D	3,0 (1,1;7,7)	90,2 (80,0;95,5)	6,8 (3,0;14,8)	100%	
Total	4,6 (2,4;8,7)	82,0 (72,3;88,9)	13,3 (7,1;23,8)	100%	0,178

A proporção de crianças com doses coincidentes aos 4 meses foi maior entre as do estrato socioeconômico D (90,2%, IC_95_% 80,0;95,5), e as de oportunidades recuperadas, entre as do estrato A (9,9%, IC_95_% 4,2;21,7). O estrato B apresentou a maior proporção de ausência de vacinas (16,0%, IC_95_% 5,3;39,3), porém, não houve diferença estatística (p=0,178) (Tabela 4).

Por fim, com relação às vacinas previstas aos 6 meses de idade, 83,0% (IC_95_% 73,6;89,6) receberam todas as doses na mesma data, e 4,8% (IC_95_% 2,0;10,7) tiveram oportunidades recuperadas (Tabela 5). No caso destas, a vacina penta foi aplicada antes da vacina VIP em 50,0% (IC_95_% 28,2;72,0) dos casos; 2,1% (IC_95_% 1,0;4,0) das crianças apresentaram oportunidades não recuperadas, sendo a vacina VIP a de menor cobertura, ausente em 66,7% (IC_95_% 30,0;92,5) dessas. E 7,4% (IC_95_% 5,2;10,2) das crianças não receberam nenhuma das duas doses.

**Tabela 5:** Proporções (%) e intervalos de confiança de 95% (IC_95_) das carteiras de vacinação com oportunidades recuperadas, datas coincidentes ou doses ausentes referentes às vacinas aplicadas aos 6 meses, por estrato socioeconômico, em Londrina, de nascidos em 2017 e 2018 (n = 456)

Estrato	Doses aos seis meses (penta e VIP)				
	**Oportunidades recuperadas** **% (IC**_95_ **%** **)**	**Datas das** **doses coincidentes** **% (IC**_95_ **%** **)**	**Ausência** **de uma ou mais vacinas** **% (IC**_95_ **%** **)**	**Total**	**p-valor**
A	4,3 (1,9;9,5)	82,3 (72,0;89,4)	13,3 (6,5;25,4)	100%	
B	3,4 (0,5;19,8)	81,1 (58,6;92,9)	15,5 (5,0;39,2)	100%	
C	8,1 (3,3;18,9)	80,9 (73,8;86,4)	10,9 (5,0;22,4)	100%	
D	2,0 (0,6;6,8)	93,3 (82,8;97,6)	4,7 (1,3;15,8)	100%	
Total	4,8 (2,0;10,7)	83,0 (73,6;89,6)	12,2 (6,2;22,6)	100%	0,499

Diante das doses aplicadas aos 6 meses, o estrato socioeconômico D apresentou maior coincidência entre as datas das doses das vacinas aplicadas (93,3%, IC_95_% 82,8;97,6), e o estrato C expressou maior oportunidade recuperada (8,1%, IC_95_% 3,3;18,9), O estrato B apresentou a maior ausência de vacinas (15,5%, IC_95_% 5,0;39,2). Não houve significância estatística (p=0,499) (Tabela 5).

## DISCUSSÃO

Em relação às oportunidades perdidas não recuperadas de vacinação, as proporções variaram de acordo com a faixa etária observada. Os maiores valores foram encontrados nas vacinas aplicadas aos 4 meses e ao nascer, enquanto as oportunidades recuperadas, nas doses ao nascer e aos 2 meses. Não houve diferença estatística na relação entre os estratos e a regularidade vacinal.

Para as vacinas aplicadas ao nascer, a BCG apresentou maior proporção de oportunidade recuperada de vacinação. O atraso na BCG demonstra que a aplicação não está ocorrendo na maternidade, como recomendado pelo Ministério da Saúde. Como a vacina possui técnica específica e complexa para ser aplicada,^3^ a falta de capacitação técnica é uma das principais causas da não aplicação da vacina em recém-nascidos, como já apontado por um estudo realizado em Porto Alegre.^12^ Apesar disso, a maior taxa de oportunidade não recuperada é a da vacina da HepB, o que também foi encontrado em estudo com metodologia ativa em menores de 1 ano em São Paulo.^13^ A não aplicação da HepB nas primeiras 12 horas de vida é extremamente grave, pois essa janela de tempo é crucial para prevenir a transmissão vertical do vírus da mãe para o bebê, especialmente se a mãe for portadora. Quando a vacina é administrada dentro desse período crítico, ela atua como uma medida de profilaxia imediata, prevenindo a infecção antes que o vírus tenha a oportunidade de se estabelecer no organismo da criança.^3^

No que concerne às vacinas aplicadas aos 2 e 4 meses, a VRH foi a de maior proporção não recuperada. Um estudo de metodologia retrospectiva utilizando uma base de dados nacional realizado no Peru também aponta esse resultado e alerta para a tendência do crescimento desse resultado na América do Sul.^14^ A VRH é de grande importância para a saúde pública, diminuindo drasticamente as taxas de hospitalizações, complicações e mortes pela doença.^15^ A primeira dose da VRH deve ser dada até aos 3 meses e meio de idade, enquanto a segunda dose, aos 7 meses e meio, e esse limite rígido na idade de aplicação da vacina pode ser uma justificativa para o número expressivo de oportunidades perdidas não recuperadas de vacinação.^16,17^ Um estudo americano também aponta como principais causas que contribuem para a queda na cobertura da VRH a preocupação dos pais acerca da segurança geral da vacina e falhas nos locais de aplicação da vacina, além de evidenciar que a maioria das crianças não vacinadas tiveram pelo menos uma oportunidade perdida de receber a vacinação, apontando crescimento de 10% no esquema completo de vacinação contra a doença se todas essas oportunidades perdidas tivessem sido aproveitadas. ^18,19^

Em relação às vacinas aplicadas aos 6 meses, as terceiras doses da penta e da VIP não apresentaram perdas tão expressivas quando comparadas às doses anteriores. A VIP apresentou a maior proporção recuperada e não recuperada, diferentemente do encontrado em um estudo realizado em São Luis.^20^ Por outro lado, a ausência de vacinação condiz com uma tendência mundial de queda de cobertura de ambas as vacinas, e é similar ao encontrado em artigos que analisaram as taxas de vacinação nos âmbitos nacional e regional nos últimos anos.^5,21^

As oportunidades recuperadas de vacinação, apesar de permitirem que a criança receba as vacinas que perdeu, indicam que ela não recebeu as vacinas na data determinada, necessitando de mais visitas ao sistema de saúde para completar o esquema vacinal. Como podem detectar irregularidades na aplicação de determinada vacina, identificá-las é de suma importância para prevenir o surgimento de falhas na imunização da população infantil. No estudo, com exceção das doses aplicadas ao nascer, todas as idades tiveram a vacina de maior oportunidade recuperada como também a de maior oportunidade perdida. Ainda, elas não implicam necessariamente doses válidas de vacinação, já que cada vacina apresenta um período mínimo no qual deve ser aplicada para melhor resposta imunológica.^3^

As causas relatadas de oportunidades perdidas de vacinação atualmente são variadas e comuns em vários artigos publicados. Em estudos realizados na Cidade do Cabo (Nigéria), Malawi e Chade, utilizando questionários aos cuidadores e verificação de carteira vacinal nas saídas das unidades de saúde, os números de oportunidades perdidas de vacinação encontram-se muito superiores aos encontrados em Londrina, chegando a taxas de quase 40%.^22-24^ Utilizando como população crianças de 0 a 23 meses, reforçam a grande proporção de incompletude vacinal em menores de 1 ano, apontando como principais causas a ausência de checagem das carteiras de vacinação, a falta de conhecimento e capacitação dos trabalhadores da área da saúde acerca do assunto, desconhecimento dos pais sobre a importância das vacinas e das oportunidades perdidas de vacinação, e problemas nas campanhas vacinais e na distribuição e oferta de vacinas. Tais causas coincidem com aquelas apontadas pelo estudo realizado no Peru,^14^ que acrescenta o medo de efeitos adversos e a recusa de grande quantidade de vacinas aplicadas simultaneamente. Esta última, inclusive, pode estar associada às oportunidades recuperadas. Segundo um trabalho argentino,^25^ a utilização plena de todas as oportunidades para a administração simultânea de todas as doses de vacinas elegíveis para a idade, durante a mesma visita de vacinação, é uma estratégia crucial para alcançar a meta de cobertura vacinal. Assim, é essencial capacitar os trabalhadores da área da saúde tanto para a aplicação de vacinas, como para verificar as carteiras de vacinação das crianças, em qualquer contato destas com os serviços de saúde, abordando também as vacinas faltantes.

 A literatura evidencia que a classe socioeconômica, o nível de alfabetização dos cuidadores e fatores culturais influenciam diretamente a adesão ao calendário vacinal infantil.^4,10,25,26^ A estratificação socioeconômica foi vantajosa para representar adequadamente os diferentes setores censitários de Londrina, oferecendo uma visão clara das desigualdades vacinais.

O estrato socioeconômico D – ou seja, crianças que vivem nos setores censitários mais desfavorecidos – apresentou as maiores proporções de datas coincidentes entre as doses recebidas nas respectivas idades quando comparado aos outros estratos, enquanto os estratos A e C, as menores ausências de vacinas. Entretanto, não houve diferença estatística entre os estratos. 

Em pesquisas em capitais brasileiras, crianças de estratos socioeconômicos mais elevados registraram a menor taxa de vacinação completa e menor cobertura para várias vacinas individuais,^26^ demonstrando uma tendência contemporânea da queda de cobertura que se relaciona com os estratos mais ricos. Assim, sugere-se que os programas de imunização do serviço público, que é o mais utilizado pela população mais desfavorecida, estão cumprindo com as recomendações do PNI, e que provavelmente as perdas vacinais não estejam relacionadas com a estrutura desses locais de aplicação.

A diminuição, ao longo dos últimos 40 anos, da ocorrência de doenças imunopreveníveis atrelada a ondas crescentes de informações falsas sobre os efeitos adversos da vacina pode gerar uma atitude de desinteresse e hesitação entre os segmentos mais privilegiados da população londrinense, como já apontado em países desenvolvidos e no Brasil.^27-29^ Esse cenário propicia o descumprimento do calendário vacinal infantil entre os estratos socioeconômicos altos, causando taxas de perdas vacinais. Como boa parte dessas crianças tendem a utilizar serviços privados de vacinação, é importante entender a dinâmica adotada pelos profissionais de saúde, a fim de minimizarem a hesitação dos cuidadores que impedem que as crianças recebam todas as vacinas necessárias em dada sessão. Dessa forma, é imprescindível que haja, por parte do poder público, a identificação das principais barreiras que dificultam, para a população dos estratos altos, a regularização do esquema vacinal de suas crianças, e o direcionamento de campanhas educativas que desmistifiquem os medos desse segmento da população. Ademais, é necessário envolver o sistema privado de saúde no combate às oportunidades perdidas de vacinação.

O estudo engloba uma distribuição significativa das crianças da área urbana de Londrina, possuindo uma amostra representativa do local, além de não ter apresentado perda amostral. Apesar disso, como limitação, não abarcou a área rural, que possui população de 13.181 crianças.^30^ Ainda, a leitura da caderneta de vacinação também pode conter erros, dada a falta de padronização de anotações e datas ilegíveis, dificultando a transcrição de dados. 

O estudo revela perdas vacinais para vacinas que deveriam ser aplicadas na mesma sessão. Recomendam-se, portanto, campanhas educativas focadas na desinformação e medo dos efeitos adversos, capacitação dos profissionais da sessão de vacinação e melhor integração entre os sistemas público e privado de saúde, a fim de se garantir o monitoramento contínuo do estado vacinal. Estudos futuros devem explorar as causas da hesitação vacinal e estratégias para reduzir essas oportunidades perdidas, visando maior adesão ao calendário vacinal.
